# An integrative approach to enhancing small-scale poultry slaughterhouses by addressing regulations and food safety in northern -Thailand

**DOI:** 10.1186/2049-9957-3-46

**Published:** 2014-12-05

**Authors:** Suwit Chotinun, Suvichai Rojanasthien, Fred Unger, Manat Suwan, Pakpoom Tadee, Prapas Patchanee

**Affiliations:** Department of Food Animal Clinics, Faculty of Veterinary Medicine, Mae Hia, Muang, Chiang Mai, 50100 Thailand; International Livestock Research Institute (ILRI), Nairobi, Kenya; Department of Geography, Faculty of Social Sciences, Chiang Mai University, Chiang Mai, Thailand

**Keywords:** Integrative approach, Hygienic practices, Regulation, Small-scale poultry slaughterhouse

## Abstract

**Background:**

In Asian countries, small-scale rural poultry meat production can face challenges due to food safety policies that limit economic growth and hinder improvement of sanitation and disease prevention. In this study, an integrative, participatory research approach was used to elucidate the sanitation and disease prevention practices in small-scale poultry slaughterhouses in rural northern Thailand.

**Methods:**

Initial steps included the identification of key stakeholders associated with the meat production chain, development of a research framework, and design of a methodology based on stakeholder consultations. The framework and methodology combine issues in five major areas: (1) public health, (2) socioeconomics, (3) policy, (4) veterinary medicine, and (5) communities and the environment. Methods used include questionnaires, direct observation, focus groups, and in-depth interviews. In addition, a microbiological risk assessment approach was employed to detect *Salmonella* contamination in meat processing facilities. The microbial risk assessment was combined with stakeholder perceptions to provide an overview of the existing situation, as well as to identify opportunities for upgrading slaughterhouses in order to more effectively address matters of food safety, processing, and government licensing.

**Results:**

The conceptual framework developed elucidated the complex factors limiting small-scale slaughterhouse improvement including a lack of appropriate enabling policies and an apparent absence of feasible interventions for improvement. Unhygienic slaughterhouse management was reflected in the incidence of *Salmonella* contamination in both the meat and the surrounding environment.

**Conclusion:**

There is potential for the use of an integrative approach to address critical problems at the interface of rural development and public health. The findings of this study could serve as a model for transdisciplinary studies and interventions related to other similar complex challenges.

**Electronic supplementary material:**

The online version of this article (doi:10.1186/2049-9957-3-46) contains supplementary material, which is available to authorized users.

## Multilingual abstracts

Please see Additional file 1 for translations of the abstract into the six official working languages of the United Nations.

## Background

Interest in an integrative approach has been increasing, especially in the area of ecosystem health, in response to growing evidence that many important ecosystem changes are the result of human activity [1]. The current environmental imbalance is considered to be a factor contributing to the outbreak of emerging diseases, as well as re-emerging diseases such as severe acute respiratory syndrome (SARS), hantavirus, highly pathogenic avian influenza, and other foodborne diseases. Although traditional approaches involving medical technology combined with active involvement of social, ecological, and political disciplines can be an effective tool in controlling diseases [2], there have been numerous outbreaks of SARS and other diseases over the last three decades indicating a need for additional methods.

Food safety is an issue which is related to ecosystem health that concerns all human beings, making it an important focus of public health strategies around the world, including Thailand. Cases of foodborne diseases are still increasing, especially in developing countries, so food safety remains a serious challenge.

*Salmonella* is one of the most common organisms causing foodborne diseases worldwide. In the US, *Salmonella* is the second largest cause of food poisoning, where it spread widely between 2009 and 2010 [3]. In EU countries, approximately 100,000 patients suffered from food poisoning caused by *Salmonella* in 2010. In Thailand, *Salmonella* was found to be the second largest cause of food poisoning, following rotavirus in 2008 [4]. The main cause of human salmonellosis is the consumption of meat such as chicken contaminated with *Salmonella* [5]. Processing procedures in poultry slaughterhouses have been identified as an important source of *Salmonella* contamination of chicken meat, [6] especially in small-scale poultry slaughterhouses where traditional slaughtering processes are common [4].

To enhance food safety and control foodborne diseases, efforts have been made to enforce regulations specifying standards for poultry slaughterhouses in Thailand, however, many small-scale slaughterhouses have yet not been able to meet these standards [7]. This study aimed to identify problems related to food safety in poultry production and to develop a conceptual framework for elucidating the administration situation and the potential for enhancement of hygienic management of small-scale poultry slaughterhouses in northern Thailand to help them achieve compliance with standard regulations.

## Methods

### Conceptual framework development

Since food safety, especially in rural areas of Thailand, is a complex challenge and involves many sectors, an integrated approach was applied in this study. The main stakeholders were identified in the early stages of the project, using participatory methods, e.g., researchers meeting with key stakeholders including slaughterhouse owners, as well as Department of Livestock Development (DLD) officers at the national and regional levels, to identify problems. The information obtained from discussions with stakeholders was then reviewed with experts in veterinary science, socioeconomics, and public health before being used as the basis for developing a conceptual framework.

### Current laws and regulations

Policies, laws, and regulations which include the key phrases “food safety”, “slaughterhouse standards”, “current situation of poultry slaughterhouses”, or “foodborne diseases in Thailand” were collected from published and unpublished sources including the *Royal Thai Government Gazette*, the Eleventh National Economic and Social Development Plan of Thailand (2012–3016), the DLD strategic plan, as well as domestic and international research reports on poultry slaughterhouses.

### Implementation of laws and regulations

Perceptions regarding the implementation of existing regulations were obtained through focus group discussions (FGDs) with DLD regional officers. The principle investigator and co-principle investigator led the FGDs. Purposive sampling was used to identify participants using the criteria: (1) DLD provincial officers; (2) heads of DLD district offices; and (3) individuals having responsibly for slaughterhouse control. Two FGDs were conducted with a total of 22 participants between May and June 2012. Qualitative content analysis was used to analyze FDG data using the five-step process as described by Agus et al. [8]: (1) Following transcription of the interviews, summaries of the discussions were compiled; (2) All interviews were coded and categorized, outlined, then grouped under appropriate headings; (3) Similar headings were combined and categories were generated to reflect the study aims; (4) Analysis of the trustworthiness of the results was performed by asking a colleague to generate a theme list; and (5) Each transcript was coded by theme.

### Study site and data collection

The Chiang Mai province is located in the northern part of Thailand and is characterized as having dense areas of poultry production. In 2010, more than three million chickens were produced in this province [9]. For that reason, Chiang Mai was selected to be the focal site of this study. A total of 41 small-scale poultry slaughterhouses (each processing fewer than 50 birds/day) were visited during the period from July 2011 to May 2012. Data on the current status of the slaughterhouses, especially data regarding productivity, economic status, hygienic management, and opportunities and challenges faced in improving the plants and following the DLD slaughterhouse regulations, were collected using a structured questionnaire and interviews (see Additional file 2). In addition, a checklist, which was developed based on the DLD regulations, was used for triangulation (see Additional file 3). The data were analyzed by way of descriptive statistics using the Microsoft Excel 2010 program (Microsoft Corp.).

### Sample collection and Salmonella identification

Evidence of the prevalence of *Salmonella* spp. was used to elucidate the public health and environmental hazards of poultry meat production. A study by Padungtod and Kaneene found a 9% incidence of *Salmonella* contamination in meat processed by slaughterhouses in northern Thailand [10]. Based on that data, this study used a 10% expected prevalence. Sample size was computed using the Epi Info™ program with a 3% confidence limit and a 95% confidence level.

A preliminary survey found that, in 2010, Chiang Mai had 55 small-scale poultry slaughterhouses with approximately 25,000 birds being sent to these slaughterhouses each day. Samples were collected from slaughterhouses located within 100 kilometers of the laboratory at Chiang Mai University to insure that samples could arrive there within three hours. A total of 410 meat samples from 41 slaughterhouses were collected. Each carcass was placed in a large bag with 250 ml of sterile peptone water which was then shaken inside the bag for one minute, then the rinse water was poured into a sterile bottle and used for identification of *Salmonella* spp. In addition, environmental samples, including 500 grams of soil taken from around slaughterhouse buildings, were collected and stored in sterile plastic bags. In addition, one-liter samples of wastewater were collected using sterile bottles before the water was drained into the environment. Sample collection was conducted from July 2011 to May 2012.

All samples were collected in the morning immediately after completion of the slaughtering process, put into single use zip lock plastic bags, kept on ice in an ice chest, and sent within three hours of collection to the Diagnostic Center, Faculty of Veterinary Medicine, Chiang Mai University, for testing for the presence of *Salmonella* spp. The cold chain was not broken during sample collection and transport to the Diagnostic Center. Scientists used a standard Diagnostic Center form to record information on each ice chest including the number of the ice chest, the owner of the slaughterhouse, the sender of the sample, and individual sample identification information. Samples were then stored in a refrigerator at 4°C prior to individual sample testing which were conducted the following morning. After each use, each ice chest was washed with dishwashing liquid and water and then dried in a plate dryer. To further preclude possible contamination, each ice chest was withdrawn from use for between five and seven days after delivering the samples. The corresponding author (DVM, MS Health Science) supervised sample collection in the field. The Diagnostic Center is certified by the Bureau of Laboratory Quality Standard (BLQS), Department of Medical Sciences, Ministry of Public Health.

*Salmonella* identification was performed according to a modified version of the US Food and Drug Administration *Salmonella* culture method (*Bacteriological Analytical Manual*) [11], and ISO 6579: 2002 and 2007 with Annex D [12]. The prevalence of *Salmonella* contamination in carcasses, soil, and wastewater was calculated by dividing the number of samples positive for *Salmonella* by the total number of samples processed. Concurrent with the sample collection, data on the characteristics of the slaughterhouses, including slaughterhouse management and perceptions of the owners regarding slaughterhouse standard regulations, were collected by the principle investigator at the slaughterhouses using structured questionnaires and interviews. The questionnaire focused on processing capacity, biosecurity, disease control management, veterinary services, health status of workers, environmental management procedures, socioeconomic situation, and perception of zoonotic aspects.

In addition, a checklist of DLD regulations regarding the location of the facility, characteristics of the structures, the slaughtering process, and waste management was applied as a cross-check of the data. Descriptive statistical analysis was accomplished using Microsoft Excel 2010 (Microsoft Corp.).

## Results

### Conceptual framework and problem identification

The main stakeholders in this study were the slaughterhouse owners, DLD officers at the national and regional levels, regional public health officers, and local administration officers. Brainstorming meetings and interviews confirmed the stakeholders’ views on the importance of food safety and food policies in Thailand. They realized that poultry slaughterhouses are an important link in the poultry meat production chain, that the standard regulations should be followed, and that there are many factors affecting the improvement of slaughterhouses. They concurred that the main problems to be addressed are the inability of most small-scale slaughterhouses to comply with the current standard regulations and a lack of appropriate strategies to motivate and assist small-scale slaughterhouses to comply with these regulations.

**Figure 1:**
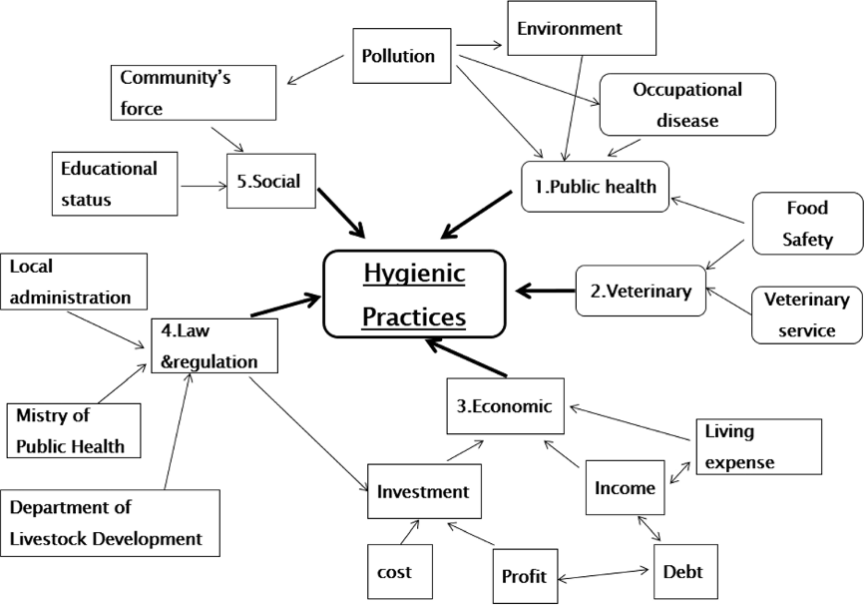
**The conceptual framework of the study.**

The conceptual framework of this study (see Figure 1) reveals the complex interactions related to achieving slaughterhouse improvements. For example, there are three main government agencies responsible for the control of slaughterhouses: (1) the DLD, which is primarily responsible for animal health and disease control on livestock farms plus improvement and updating of regulations governing slaughterhouses; (2) The Ministry of Public Health, which is responsible for setting food safety standards for meat products; and (3) Local administrative organizations, which are responsible for giving permission to slaughter animals and to distribute meat, as well as appointing meat inspectors. To effectively assist slaughterhouse owners to improve their slaughterhouses and to follow regulations, government officers from these agencies must work together in an integrative mode.

The framework also includes socioeconomic factors affecting the improvement of slaughterhouses, for example, the association of education level and age with perceptions of food safety, as well as issues of income from slaughterhouses, living expenses, and family debt that could affect opportunities for investment in slaughterhouse improvements.

### Laws and regulations governing slaughterhouses in Thailand

The main regulation regarding slaughterhouse control is the Ministerial Regulation on Determination of Criteria, Procedures and Conditions for Establishing Slaughterhouses, Lairage and Animal Slaughter B.E. 2555 (2012) [13]. This regulation consists of seven topics: (1) the location of the slaughterhouse, e.g., slaughterhouses must be situated far away from communities); (2) the area and structure of the slaughterhouse buildings, e.g., the slaughtering process must be conducted in a concrete building and there must be a fence around the slaughterhouse; (3) local infrastructure and the area inside of the slaughterhouse, e.g., the area inside the slaughterhouse building must be appropriate for operations, easy to clean, and include separate clean and dirty zones; (4) equipment and facilities management, e.g., facilities used in the slaughtering process must be easy to clean; (5) holding pens, e.g., pens where birds are maintained for 8–10 hours before slaughter must be constructed of concrete and must prevent pathogens contamination of the slaughtering process; (6) waste management systems; and (7) hygiene management, e.g., cleaning the slaughterhouse every day after operation. To be licensed by the DLD, all slaughterhouses in Thailand must comply with this regulation.

In addition, good manufacturing practices (GMPs) for poultry slaughterhouses were announced, and their adoption has been mandatory since 2006. However, in practice, the GMP guidelines, which were intended to further improve operations including hygiene standards, have been enforced only in slaughterhouses which have been issued a government license, most of which are larger operations.

### Implementation of standard slaughterhouse regulations

In the FGDs with DLD regional officers (provincial and district) on the implementation of laws and regulations, the officers accepted that they could not strictly enforce the ministerial regulatory criteria intended to promote the improvement of small-scale slaughterhouses. They acknowledged that the criteria are intensive and require high levels of investment, making them suitable for large- and medium-scale operations which generate sufficient profit, but not for small-scale facilities with low productivity and small profits. They agreed that if they attempted to strictly enforce the regulations, they would meet resistance from the slaughterhouse owners. They also acknowledged that during their regular visits to slaughterhouses every three to four months, they should focus on establishing a spirit of collaboration and cooperation in order to promote hygienic management and disease control in slaughterhouses rather than strictly enforce the regulations. They also indicated that the current regulations should be more flexible and practical. As one officer said, “It would be useful if there was a prototype or a blueprint of a good, hygienically managed slaughterhouse that owners could use as a model for investment”. The majority of the participants agreed with this comment.

Department of Livestock Development officers indicated an awareness of the need to work integratively with officers from other agencies, including public health officers and local administrative officers, in order to improve food safety. However, they mentioned that there were obstacles to such joint efforts. For example, working with local administrative organizations was problematical because those organizations still had no official role in that area or any personnel specifically responsible for slaughterhouse control. In the case of Public Health agencies’ work with food safety control, their main focus is on meat products sold in the market rather than conditions at slaughterhouses. On a positive note, just over half the participants (54.5%) indicated that they were willing to work in an integrative manner with other agencies to address issues of food safety. Table 1 summarizes the reflections of the participants regarding the themes of the FGDs.

**Table 1:** **Reflections of DLD officers from FGDs**

***FGD topics***	***Agreement (%)***
Current regulation is suitable and practical for small-scale poultry slaughterhouse	0.0
Current regulation is only suitable for large- and medium-scale poultry slaughterhouse	77.3
The officer could effectively enforce the regulation	0.0
The current regulation should be flexible and practical for a small-scale slaughterhouse	72.3
Blueprint of well-managed small-scale facilities is very useful	90.9
DLD officers have problem of working with other associated officers to improve the slaughterhouses	68.2
DLD officers still have to carry out integrative work with associated officers to improve food safety	54.5

### Slaughtering processes and hygienic practices

Of the participating slaughterhouse owners (see Table 2), 46.3% were male; 48.8% were 50–59 years old; 68.3% had completed primary school; 24.4% had been operating a slaughterhouse for 11–15 years; 82.9% slaughtered 1–50 birds/day; and 100.0% did not have a DLD license for slaughtering.

**Table 2:** **Characteristics of participating slaughterhouse owners in the study**

***Characteristics***	***Number***	***Percentage***
Gender		
*Male*	19	46.3
*Female*	22	53.7
Age group (in years)		
*21–29*	1	2.4
*30–39*	9	22.0
*40–49*	8	19.5
*50–59*	20	48.8
*>60*	3	7.3
Education		
*No education*	1	2.4
*Primary*	27	65.9
*Secondary*	10	24.4
*Diploma*	2	4.9
*Bachelor*	1	2.4
Years of slaughterhouse operation		
*1–5*	9	22.0
*6–10*	9	22.0
*11–15*	10	24.4
*16–20*	6	14.6
*21–25*	4	9.7
*>25*	3	7.3
Productivity (birds/day)		
*1–50*	34	82.9
*51–100*	4	9.7
*101–150*	2	4.9
*151–200*	0	0.0
*>200*	1	2.4
License for slaughtering		
*Yes*	0	0.0
*No*	41	100.0

The process of slaughtering was carried out in open-air buildings as follows: the birds were killed with a sharp knife, and the carcasses were scalded in a water tank at a temperature of 50–70°C for 2–3 minutes. De-feathering was done using semi-automatic de-feathering machines. The carcasses were cleaned by dipping them in a bucket of water. Evisceration was done by hand, using a knife to cut open the carcasses; this process was carried out on chopping blocks placed on the floor. The carcasses were then dipped in hot water (50–70°C) to firm up the skin, then stored in a small vessel containing ice. The wastewater from the slaughtering process was discharged directly onto the area around the slaughterhouses.

The majority of the small-scale slaughterhouses, which processed fewer than 50 birds/day, did not satisfy all of the seven criteria described in the slaughterhouse law and regulation. The owners constructed simple facilities with only necessary equipment and located within their community. Birds were sold only in the local community the same day they were slaughtered, but quantities were small, just enough to meet local demand. Incomes were limited and not sufficient to invest in improvements to the slaughterhouses to meet the Ministerial criteria. Results of the slaughterhouse assessment and the hygiene management evaluation are presented in Tables 3 and 4, respectively.

**Table 3:** **Results of the assessment of the slaughterhouses’ location and structure (N = 41)**

***Criteria***	***Suitable***	***Unsuitable***	***Not implemented***
1. The slaughterhouse is not located in a community	0	41	–
2. The condition of the area outside the slaughterhouses (e.g., fenced and clean area)	1	40	
3. The structure of the building (e.g., concrete with good ventilation)	2	39	–
4. The condition of the area inside the building (e.g., separate dirty and clean areas)	2	39	–
5. Equipment and facilities (e.g., easy to clean)	1	40	–
6. Holding pen exists	1	2	38

**Table 4:** **Results of assessment of sanitary management of slaughterhouses (N = 41)**

***Criteria***	***Suitable***	***Unsuitable***
1. Proper slaughter process (e.g., having live poultry and meat inspection)	2	39
2. Equipment and facilities cleaned daily before and after operation	2	39
3. Vehicles cleaned before and after transportation of carcasses	1	40
4. Pest control program	3	38
5. Use only chemicals approved by FDA	2	39
6. Chemicals used in slaughterhouses properly stored	3	38
7. Proper waste management	7	34
8. Adequate number of clothing changing rooms, cleaning rooms, and toilets	1	40
9. Proper methods of elimination of carcasses not suitable for human consumption	1	40

### Slaughterhouse owners’ perceptions regarding slaughterhouse improvement

Regarding slaughterhouse owners’ perceptions of relevant laws and regulations, 33 out of 41 owners (80.5%) stated that some of the criteria in the current standard regulation were impractical for small-scale slaughterhouses. For example, meat from most of the smaller slaughterhouses was not inspected because the limited slaughterhouse income was not sufficient to hire a meat inspector. Owners stated that they would have to stop operating their business if the DLD strictly enforced all the standard slaughterhouse regulation requirements. Although 25 out of 41 owners (61%) accepted that they did need to improve their slaughterhouses, they indicated a desire that the regulatory criteria be more practical.

### Salmonella prevalence in carcasses and the environment

*Salmonella* spp. were isolated from 30 out of 410 carcass samples (7.3%) taken during this study. As for contamination in the environment, 12 out of 41 soil samples (29.3%) and nine out of 41 wastewater samples (21.9%) tested positive for *Salmonella* spp.

## Discussion

Over the last 30 years, several successful attempts have been made to control various infectious diseases in countries all over the world, especially in developed nations. However, threats still exist such as antimicrobial resistant bacteria and unsafe farming and food production practices, as well as threats created by the impact of urbanization and agricultural intensification [2]. In addition, traditional methods of controlling infectious diseases using conventional biomedical strategies have often failed, resulting in the emergence and outbreak of diseases such as SARS, H5N1 and H7N1 avian influenza, malaria, tuberculosis, etc. [14]. In order to address these challenges and to achieve improvements in overall health—not just human health—the crucial roles of social, economic, and cultural factors must also be considered. Thus it is imperative that non-medical sciences be involved in the process of developing disease control strategies.

To more effectively identify avenues for enhancing safe processing in small-scale poultry slaughterhouses, practitioners of veterinary and human medicine, social scientists, and economists cooperatively followed an integrative approach in the development of the conceptual framework and in participatory problem identification from the outset. That framework demonstrates the complexity of the problem and the linkages between the different disciplines. This study follows the successful integrative approach which was used to gain an understanding of and develop a suitable research agenda in the case of the emergence of leptospirosis in Hawaii [15]. This study evidences the importance of a transdisciplinary approach, as well as methods of implementing that approach as described and demonstrated by Pokras and Kneeland in their development of educational and policy initiatives to control the lead poisoning problem in wildlife, humans, and domestic animals [16].

This study confirms that good hygienic management is not widely practiced in small-scale slaughterhouses in northern Thailand. This finding is consistent with the report in Bangladesh by Rimi et al. in 2013, which reported commonly observed improper practices such as slaughtering sick poultry in rural communities [17]. This study shows that, in general, hygienic practices do not fully follow existing regulations and that the government provided guidelines are not implemented by many slaughterhouses, e.g., the slaughtering process being performed on the floor, and the lack of methods or processes to prevent bacterial contamination of carcasses.

The majority of slaughterhouse owners pointed out that they could not improve their operations in accordance with the current standard regulation. For instance, the regulation specifies that slaughterhouses must not be located in communities, a challenge to the small-scale slaughterhouse owners in this study currently located within a community. In fact, some of the slaughterhouses were in operation prior to the regulation. An authorized meat inspector is required to inspect the meat, but owners pointed out that they could not afford to hire a meat inspector because of the limited profitability of their operations. Owners stated further that if the DLD decided to strictly enforce the regulation, they would have to cease operation of their slaughterhouse and seek a new occupation, an outcome that would certainly adversely affect the socioeconomic status of the owners and their families.

Department of Livestock Development regional officers agreed that the current laws and regulations are, in fact, more suitable for large- and medium-scale operations which can afford the necessary high investment. Nonetheless, the DLD is attempting to encourage even small-scale poultry slaughterhouses to meet the standard. The slaughterhouse blueprint developed by the DLD and distributed to officers and slaughterhouse owners, however, is designed for operations processing 200–300 birds/day which is four or more times the daily production of small-scale slaughterhouses. Thus, the DLD-proposed blueprint poses a considerable challenge to small-scale slaughterhouses.

The importance of *Salmonella* as a public health hazard was clearly demonstrated in this study, which found a prevalence of 7.3% in the final product (chicken carcasses). That figure is close to the 9% prevalence of *Salmonella* in poultry carcasses after slaughtering and final products in Thai slaughterhouses reported in a study by Padungtod and Kaneene in 2006 [16], but much lower than in other studies, e.g., Kueylaw et al. in 2008 [7] found a prevalence of *Salmonella* of 43%. Reports from elsewhere in the world also indicate a higher prevalence of *Salmonella*. For example, Elgroud et al. reported in 2009 that the prevalence of *Salmonella* in chicken slaughterhouses in Algeria was over 53% [18], while Fuzihara et al. reported in 2000 a 42% prevalence of *Salmonella* in chicken carcasses from small-scale poultry slaughterhouses in Brazil [19]. Similarly, Bohaychuk et al. reported in 2009 that the prevalence of *Salmonella* in poultry slaughterhouses in Alberta City, Canada was 37% [20], while Capita et al. found in 2006 that the prevalence of *Salmonella* in chickens from slaughterhouses in Spain was 17.9% [21].

*Salmonella* prevalence in these reports is significantly higher than that found in this study. One possible reason for the lower *Salmonella* prevalence found in the current study could be that the survey was conducted at small-scale facilities, the majority of which processed fewer than 50 birds/day. Processing fewer birds might result in a lower bacterial load in those facilities and thus a lower *Salmonella* prevalence in carcasses compared with other studies such as the one by Padungtod and Kaneene (2006), which was done in medium- and large-scale slaughterhouses. Moreover, the traditional slaughtering process commonly found in smaller operations includes the final processing step of immersing the carcass in hot water for a short time to firm the skin. Immersion makes the skin more attractive, an important factor for small-scale operations which sell the final product (carcasses) in the local community. That process also has the effect of decreasing pathogen contamination. However, this method could also have the negative effect of increasing the temperature of the carcasses, making them more suitable for bacterial growth and thus more susceptible to rotting. For that reason, it is not appropriate for carcasses treated this way to be stored overnight.

The 29.3% prevalence of *Salmonella* contamination found in soil collected around slaughterhouse buildings and the 21.9% contamination rate in wastewater drained onto the area around the slaughterhouse without treatment, however, is evidence that improper hygienic practices can affect not only end consumers, but also members of the local community and the surrounding environment. These results mirror findings in previous studies in other regions of the world. For example, 100.0% of sludge samples collected from eight pig and five poultry slaughterhouses in Belgium and the Netherlands were found to be contaminated with *Salmonella* [22], and 7.4% of treated effluent samples from seven pig and seven poultry slaughterhouses in Brazil taken in 2003–2004 were positive for *Salmonella* spp. [23]. Seven out of 22 samples (31.8%) obtained in 1993 from untreated wastewater from Nigerian slaughterhouses and river water collected at sites near those slaughterhouses tested positive for *Salmonella* [24]. Thus, it can be inferred that slaughterhouses are a potential source for dissemination of foodborne pathogens into the environment, especially where poorly treated or untreated wastewater is discharged directly into the environment.

## Conclusion

Improving small-scale poultry slaughterhouses to meet food safety standards and addressing the standard certification requirement are challenges that need to be addressed. This study, which found that unsatisfactory and inadequate management of hygiene was common in small-scale poultry slaughterhouses in Thailand, demonstrates that the use of an integrative approach for exploring a complex problem and developing a research conceptual framework can be an effective approach. Even though the level of *Salmonella* contamination found in carcasses was low compared with some previous studies, hygiene management should be improved to enhance food safety. Current laws and regulations are not suitable for most small-scale slaughterhouses in Thailand as few have the capacity to conform to the existing regulations. It is suggested that the potential health hazards to the community from *Salmonella* and other foodborne diseases emanating from small-scale poultry slaughterhouses should be studied further. In addition, specific regulatory criteria which meet appropriate food safety standards but which are specifically designed to be suitable and practical for small-scale slaughterhouses should be developed, pilot tested in the field, and then implemented.

## Electronic supplementary material

Additional file 1: **Multilingual abstracts in the six official working languages of the United Nations.** (PDF 239 KB)

Additional file 2: **Questionnaire for Slaughterhouses.** (DOC 181 KB)

Additional file 3: **Check list for slaughterhouses.** (DOCX 17 KB)
