# Supplementary material for: An integrative approach to enhancing small-scale poultry slaughterhouses by addressing regulations and food safety in northern -Thailand
Source: Infect Dis Poverty. 2014 Dec 5;3:46. doi: 10.1186/2049-9957-3-46 (PMC4322817; doi:10.1186/2049-9957-3-46)
Supplement: Supplementary file 1 — Additional file 1: Multilingual abstracts in the six official working languages of the United Nations. (PDF 239 KB) [file 40249_2014_86_MOESM1_ESM.pdf]

Translation of the abstract into the six official working languages of the United Nations

نهج تكاملي لدعم مجازر الدواجن صغيرة النطاق من خلال معالجة اللوائح وسلامة الأغذية في شمال تايلاند

باتشاني تادي، وبراباس سوان، باكبوم روجاناستين، فريد أنجر، مانوت سويت تشوتينون، سوفيتشاي

ملخص
في البلدان الآسيوية، تواجه مجازر لحوم الدواجن الريفية الصغيرة تحديات نتيجة لسياسات السلامة خلفية:
في هذه الغذائية التي تحد من النمو الاقتصادي وتعيق تحسين الإجراءات الصحية والوقاية من الأمراض.
الدراسة، تم استخدام منهج البحث بالمشاركة التكاملي لتوضيح ممارسات الإجراءات الصحية والوقاية من
الأمراض في مجازر الدواجن صغيرة النطاق في المناطق الريفية في شمال تايلاند.
وتضمنت الخطوات الأولية تحديد أصحاب المصلحة الرئيسيين المرتبطين بسلسلة إنتاج اللحوم، ووضع إطار
البحث، وتصميم منهجية تقوم على التشاور مع أصحاب المصلحة. الإطار والمنهجية تجمع بين قضايا خمسة
الطب البيطري (4)السياسة (3)الاوضاع الاقتصادية الاجتماعية (2)الصحة العامة (1) مجالات رئيسية هي:
وتشمل الأساليب المستخدمة الاستبيانات والملاحظة المباشرة، ومجموعات التركيز، (5) المجتمعات والبيئة.
بالإضافة إلى ذلك، استُخدم نهج تقييم المخاطر الميكروبيولوجية للكشف عن تلوث والمقابلات المتعمقة.
وتم الجمع بين تقدير المخاطر الميكروبية مع تصورات أصحاب المصلحة السالمونيلا في مرافق تجهيز اللحوم.
لتقديم لمحة عامة عن الوضع القائم، فضلا عن تحديد الفرص المتاحة لتطوير المجازر من أجل معالجة أكثر
فعالية فيما يتعلق بمسائل سلامة الأغذية وتجهيزها والتراخيص الحكومية.
الإطار المفاهيمي الموضوع يوضح العوامل المعقدة التي تحد من تحسين أوضاع المجازر الصغيرة النتائج:
وقد تسبب الحجم بما في ذلك عدم وجود سياسات تمكينية مناسبة وغياب واضح للتدخلات الممكنة للتحسين.
إدارة المجازر بطريقة غير صحية في حدوث تلوث السالمونيلا في كل من اللحوم والبيئة المحيطة.
هناك إمكانية لاستخدام نهج تكاملي لمعالجة المشاكل الحرجة في واجهة التنمية الريفية والصحة الاستنتاج:
نتائج هذه الدراسة يمكن أن تكون نموذجا للدراسات العبرمناهجية والتدخلات المتعلقة بالتحديات المعقدة العامة.
الأخرى المماثلة.

Translated from English version into Arabic by Mahmoud Sami, through

TRANSLATORS
WITHOUT BORDERS

泰国北部通过明确相关法规和食品安全以提高小规模家禽屠宰场卫生状况的一个综合评价方法

Suwit Chotinun, Suvichai Rojanasthien, Fred Unger, Manut Suwan, Pakpoom Tadee, and Prapas Patchanee

摘要

引言：在亚洲国家，农村地区的小规模禽肉生产面临很多食品安全政策相关的挑战，如限制当地经济增长，阻碍卫生状况改善和不利于疾病预防。在本研究中，使用一个综合性、参与性的研究方法分析泰国北部农村地区的小规模家禽屠宰场的卫生状况及疾病预防措施。

方法：首先进行肉类生产链关键利益相关者的鉴定，研究框架的构建和一种基于利益相关者协商方法的设计。研究框架和方法学相结合涉及 5 个方面：（1）公共卫生、（2）社会经济学、（3）政策、（4）兽用药和（5）社区和环境。所用方法包括问卷调查、直接观察、焦点人群和深入访谈。此外，将微生物风险评估方法用于检测肉类加工设施的沙门氏菌污染状况。微生物风险评估结合利益相关者的认知，可对现有相关屠宰场卫生状况形成概观，并可确定升级屠宰场的时机，用于解决食品安全、加工和政府颁布许可等事项。
结果：所开发的概念框架阐明了复杂因素可限制小规模屠宰场的改善，其中包括缺乏适当的扶持政策和明显缺少用于改善屠宰场卫生现况的可行性干预手段。沙门氏菌在肉类和屠宰场周边环境的污染发生率也反映了不卫生的屠宰场管理现况。
结论：该研究结果可作为交叉学科和其他类似复杂难题干预的范例。

Translated from English version into Chinese by Zheng Qi, through

CHINESE CENTER FOR DISEASE CONTROL AND PREVENTION
NATIONAL INSTITUTE OF PARASITIC DISEASES

Approche intégrée pour l'amélioration des abattoirs de volailles à petite échelle, concernant les règlementations et la sécurité alimentaire dans le Nord de la Thaïlande

Suwit Chotinun, Suvichai Rojanasthien, Fred Unger, Manut Suwan, Pakpoom Tadee, et Prapas Patchanee

Résumé

Contexte: dans les pays d'Asie, la production rurale de viande de volaille à petite échelle peut être confrontée à des obstacles en raison des politiques de sécurité alimentaire qui limitent la croissance économique et gênent l'amélioration sanitaire ainsi que la prévention des maladies. Dans cette étude, une approche de recherche participative intégrée a été utilisée pour mettre en lumière les pratiques sanitaires et la prévention des maladies dans les abattoirs de volailles à petite échelle dans le Nord de la Thaïlande rurale.
Méthodes: les étapes initiales ont inclus l'identification des acteurs-clé associés dans la chaine de production de viande, le développement d'un programme-cadre, et la création d'une méthodologie basée sur les consultations des parties intéressées. Le programme-cadre et la méthodologie groupent les problèmes dans cinq secteurs principaux: (1) la santé publique, (2) la socio-économie, (3) la politique, (4) la médecine vétérinaire, et (5) les communautés et l'environnement. Les méthodes utilisées incluent des questionnaires, l'observation directe, des groupes de discussion, et des entretiens approfondis. En outre, une approche de l'évaluation microbiologique des risques a été employée pour détecter la contamination par la *Salmonelle* dans les établissements de traitement de la viande. L'évaluation du risque microbien a été combinée avec la perception des parties intéressées pour fournir une vue d'ensemble de la situation existante, ainsi que pour identifier les opportunités d'améliorer les abattoirs afin d'aborder plus efficacement les questions de sécurité alimentaire, de traitement, et d'autorisations du gouvernement
Résultats: Le programme-cadre conceptuel développé a mis en lumière les facteurs complexes limitant l'amélioration des abattoirs à petite échelle, y compris le manque d'une politique adéquate favorable, ainsi qu'une apparente absence d'interventions réalisables pour une amélioration. La

gestion insalubre des abattoirs s’est reflétée dans l’incidence de la contamination par la *Salmonelle,* aussi bien dans la viande que dans l’environnement proche.

Conclusion: Il y a un potentiel pour l’utilisation d’une approche intégrée pour aborder les problèmes cruciaux à l’interface du développement rural et de la santé publique. Les constatations de cette étude pourraient servir de modèle pour des études transdisciplinaires et des interventions en relation avec d’autres problèmes complexes similaires.

Translated from English version into French by Ode Laforge, through

TRANSLATORS
WITHOUT BORDERS

Интегративный подход к укреплению малых птицебоен в свете нормативных документов и безопасности пищевых продуктов в северном Таиланде

Сувит Чотинун, Сувичаи Роджанастиен, Фред Унгер, Манут Суван, Пакпум Тади, Прапас Патчани

Аннотация

Предпосылки: В странах Азии малое производство мяса птицы в сельской местности может столкнуться с трудностями из-за политики в области безопасности пищевых продуктов, что может привести к ограничению экономического роста и сдерживанию улучшения санитарных условий и профилактики заболеваемости. В настоящем исследовании рассматривается интегративный подход на основе партисипаторного исследования, используемый для выяснения санитарных условий и практических мер по профилактике заболеваемости на малых птицебойнях в сельской местности северного Таиланда.

Методы: На начальных шагах были выявлены ключевые участники, связанные с цепью производства мяса, выработаны рамки исследования и разработана методология на основе опросов участников. Эти рамки и методология объединяют вопросы в пяти главных областях: (1) общественное здравоохранение, (2) социоэкономика, (3) политика, (4) ветеринария и (5) сообщества и их окружение. В качестве методов использовались опросники, прямые наблюдения, фокусные группы и глубинные интервью. Кроме того, для выявления зараженности сальмонеллами на мясообрабатывющих мощностях использовался подход с оценкой микробиологического риска. Оценка микробного риска совместно с восприятием участников использовались для получения картины существующей ситуации, а также для выявления возможностей усовершенствования боен с целью более эффективного учета вопросов безопасности пищевых продуктов, их обработки и правительственного лицензирования.

Результаты: В рамках выработанной концепции выявлены сложные факторы, ограничивающие улучшение малых боен, к которым относятся отсутствие разрешительной политики и очевидное отсутствие практически заметного вмешательства с целью

улучшения. Антисанитария в управлении бойнями свое нашла отражение в случае с заражением сальмонеллами как мяса, так и окружающей среды.
Вывод: Имеется потенциал для использования интегративного подхода в отношении критических проблем на стыке между развитием сельских районов и общественным здравоохранением. Результаты настоящего исследования могут служить в качестве модели для междисциплинарных исследований и вмешательств, относящихся к другим подобным сложным проблемам.

Translated from English version into Russian by Alexander Somin, through

TRANSLATORS
WITHOUT BORDERS

Método integrador de mejora de pequeños mataderos de aves mediante el estudio de la normativa y de la seguridad alimentaria en el norte de Tailandia

Suwit Chotinun, Suvichai Rojanasthien, Fred Unger, Manut Suwan, Pakpoom Tadee y Prapas Patchanee

Resumen
Antecedentes: En los países asiáticos, la producción de carne a pequeña escala en entornos rurales se enfrenta a obstáculos causados por las políticas de seguridad que limitan el crecimiento económico y entorpecen la mejora del saneamiento y de la prevención de enfermedades. En el presente estudio, se ha utilizado un método de investigación integrador y participativo con el fin de esclarecer las prácticas de saneamiento y prevención de enfermedades llevadas a cabo en los pequeños mataderos de aves del norte de Tailandia.
Métodos: Entre los primeros pasos que se han dado se han incluido la identificación de las partes implicadas clave en la cadena de producción de la carne, el desarrollo de un marco de investigación y el diseño de una metodología basada en consultas a dichas partes implicadas. Marco de trabajo y metodología comparten cuestiones en cuatro ámbitos principales: (1) salud pública, (2) ámbito socioeconómico, (3) política, (4) veterinaria y (5) comunidades y medio ambiente. El método utilizado incluye cuestionarios, observación directa, grupos focales y entrevistas en profundidad. Además, se ha empleado un método de evaluación de riesgos microbianos para detectar contaminación por *salmonela* en instalaciones de procesado de carne. La evaluación de riesgos microbianos se ha combinado con las impresiones de las partes implicadas con el fin de facilitar una visión de conjunto de la situación existente, así como identificar las oportunidades de actualizar los mataderos para abordar cuestiones de seguridad alimentaria, procesado y licencias con más eficacia.
Resultados: El marco de trabajo conceptual desarrollado ha dilucidado los factores complejos que limitan la mejora de los pequeños mataderos, incluidos una falta de políticas de apoyo adecuadas y una aparente ausencia de intervenciones de mejora viables. La gestión poco higiénica de los

mataderos se ha visto reflejada en la incidencia de contaminación por *salmonela*, tanto en la carne como en el entorno cercano.

Conclusión: Hay potencial para la utilización de un método integrador que aborde los problemas críticos de la interrelación entre desarrollo rural y salud pública. Los hallazgos del presente estudio servirán de modelo para estudios transdisciplinares e intervenciones relacionadas con otros retos de similar complejidad.

Translated from English version into Spanish by Raquel Bentué, through

TRANSLATORS
WITHOUT BORDERS
