# Supplementary material for: An integrative approach to enhancing small-scale poultry slaughterhouses by addressing regulations and food safety in northern -Thailand
Source: Infect Dis Poverty. 2014 Dec 5;3:46. doi: 10.1186/2049-9957-3-46 (PMC4322817; doi:10.1186/2049-9957-3-46)
Supplement: Supplementary file 2 — Additional file 2: Questionnaire for Slaughterhouses. (DOC 181 KB) [file 40249_2014_86_MOESM2_ESM.doc]

**Questionnaire for Slaughterhouses**

**Name of interviewee**……………………………………………………….**Code**…………………...

**Name of interviewer**………………………………..**code of interviewer**………………………….

**Date of interview**………/…………………./……………

1. Address

| Address |  |
| --- | --- |
| Telephone |  |
| Fax |  |
| GPS profile |  |

2. Reference to other holdings or geo-physical points

| **Places** | **Name** | **Distance from slaughter house** | GPS | |
| --- | --- | --- | --- | --- |
|  |  |  | X utm | Y utm |
| Community |  |  |  |  |
|  |  |  |  |  |
|  |  |  |  |  |
| Poultry farm |  |  |  |  |
|  |  |  |  |  |
|  |  |  |  |  |
| Poultry market/wet market |  |  |  |  |
|  |  |  |  |  |
|  |  |  |  |  |
| Main road |  |  |  |  |
|  |  |  |  |  |
|  |  |  |  |  |
| Water sources |  |  |  |  |
|  |  |  |  |  |

3. Demographic data

| 1. How many family members do you have | Specify………………… | |
| --- | --- | --- |
| 1. How many members involve to the business? | O Man………………. | O Woman…………………. |
| 1. Do you hire anyone to work with you | O Yes | O No |
| 1. If yes how many people you hired | O Full time Men……………….  Women…………. | O Part time  Men……………….  Women…………. |
| 1. Respondent | O Owner | O family members |
|  | O Worker |  |
| 1. Sex of the owner | O Male | O Female |
| 1. Age | O < 20 yrs | O 21-29 yrs |
|  | O 30-39 yrs | O 40-49 yrs |
|  | O 50-59 yrs | O >60 yrs |
| 1. Educational status | O None | O Primary |
|  | O secondary | O Diploma |
|  | O Bachelor | O Higher than Bachelor |
| 1. How long for running the business | O 1-5 yrs | O 6-10 yrs |
|  | O 11-15 yrs | O 16-20 yrs |
|  | O 21-25 yrs | O > 25 yrs |

4. Capacity

| Animal | Average number of head /day | Average number of head /week |
| --- | --- | --- |
| 1. Chicken |  |  |
| 1. O Broiler |  |  |
| 1. O layer |  |  |
| 1. O Native chicken |  |  |
| 1. Other poultry (identify).... ........ ........ |  |  |
| 1. Other animals (identify)..... ........ |  |  |
| 1. Origin of the chicken | O Inside the province (%..........) | O Outside the province  (%..........) |

**5. Biosecurity**

| 1. Do you carry out a withdrawal day | O Yes | O No |  |  |
| --- | --- | --- | --- | --- |
| 1. When was the last withdrawal day? And the day before? | Specify………………………………………………………………………………. | | | |
| 1. If things are very busy, is it necessary to miss the withdrawal day | O Yes | O No |  |  |
| 1. The source of water used for cleaning | O Tap water | O Underground | O Surfaced water | O Other………  …………………. |
| 1. What is used to clean | O Disinfectant | O Detergent | O Hot water | O Other……. |
| 1. Frequency of cleaning and adequacy of cleaning | O Every day | O Specify  .................. |  |  |
| 1. Type of disinfectant | O Specify................................................................................... | | | |

**6.** Disease control management

| 1. Water sources | O Tap water | O Underground | O water ways  ………… | O other …………… |
| --- | --- | --- | --- | --- |
| 1. Share water source with community (surface water) | O Yes | O No |  |  |
| 1. Is running water available in each room? | O Yes | O No |  |  |
| 1. Treat water before use | O Yes | O No |  |  |
| 1. Method of cleaning the truck | O disinfectant bath | O disinfectant house | O disinfectant spray machine | O None |
| 1. Method of cleaning of the staff | O bathing | O Hand and foot bath | O Other | O None |
| 1. Record of people in-out SLH | O Yes | O No |  |  |
| 1. Presence of pest control | O Yes | O No |  |  |
| 1. Use only chemicals approved by FDA | O Yes | O No |  |  |
| 1. Chemicals used in slaughterhouses properly stored | O Yes | O No |  |  |

**7. Veterinary services**

| 1. Are there regular inspections of the SH | O 1 time/yr | O 2 times/yr |
| --- | --- | --- |
|  | O 1 time/month | O never |
|  | O Don’t remember | O Other…………………… |
| 1. If yes, by whom | O DLD officer | O MPH officer |
|  | O Environmental officer | O Other………………….. |
| 1. When did the last time | O Specify……………………………………………………….. | |
| 1. Can you recognize the sick birds | O Yes | O No |
| 1. If yes, how the owner/worker handle sick poultry | O Slaughter them | O Condemn |
|  | O Treatment | O Send back to farm |
|  | Other……………….. |  |
| 1. How the owner/worker handle dead poultry (abnormal dead) | O Slaughter for own consumption | O Slaughter and sell |
|  | O dispose by burying | O Dispose by burning |
|  | O throwing | O Feed to companion animal |
|  | O Others  …………….………………….. |  |
| 1. How the owner/worker handle dead poultry 2. (Normal dead) | O Slaughter for own consumption | O Slaughter and sell |
|  | O dispose by burying | O Dispose by burning |
|  | O throwing | O Feed to companion animal |
|  | O Others  …………….………………….. |  |
| 1. Is there inspection of chicken before slaughtered | O Yes | O No |
| 1. If yes, by whom? | O Vet | O Paravet |
|  | O Others | O None |
| 1. If he finds a problem what he will do? | O Condemn | O Do nothing |
| 1. Inspection of carcasses | O Yes | O No |
| 1. If he finds a problem what he will do? | O Condemn | O Do nothing |
| 1. How many are rejected per day (number and %) | Specify……………………………………………………………… | |
| 1. What are the causes of the rejection | O Rot | O The chicken die by diseases |
|  | O Contaminate with waste | O Other…………… |
| 1. What do you do with rejected carcasses (Multiple choices) | O own consumption | O sell |
|  | O dispose by burying | O Dispose by burning |
|  | O throwing | O Feed to companion animal |
|  | O Others………………….. |  |

**8**. Health status

| 1. Work hours/day | …………..hours/day | |
| --- | --- | --- |
| 1. Work day/week | …………..day(s)/week | |
| 1. Use of protective equipment | O Mask | O Glove |
|  | O Other……………… | O None |
| 1. - How you use them | O Always use | O Sometimes |
|  | O Never |  |
| 1. - Why you use them | O Avoid disease | O Followed the regulation |
|  | O Other………………….. |  |
| 1. - Why you do not use them | O Not available | O Too expensive |
|  | O Too uncomfortable | O Not needed |
| 1. Condition of equipment | O Clean | O Dirty |
|  | O Intact | O Damage |
| 1. In the past month, do you have health problems | O Yes  Specify what symptoms:  - Diarrhea  - Vomiting  - Stomach pain  - Back pain  - Arm pain | O No  If NO then ask for the last 6 months |
| 1. If Yes please identify the problem | O Injury | O Respiratory problem |
|  | O Gastrointestinal problem | O Fever |
|  | O Muscle pain | O Skin problem |
|  | O Allergy | O Other…………………… |
| 1. When you got sick what did you do? | See doctor | Buy medicine |
|  | Nothing | Other……………………… |
| 1. How much days pay lost/day | O Specify…………………………………………………… | |
| 1. How much expense incurred for medicine, travel, child minding etc | O Specify…………………………………………………… | |
| 1. Have you every checked for health status | O yes | O No |
| 1. If yes when for the last time | O Specify…………………………………………………… | |
| 1. If yes how many times you check /year | O <1time/yr | O 1 time/yrs |
|  | O 2 times/yrs | O >2 times/yrs |
| 1. In general, what is your health status | O Excellent | O Good |
|  | O Fair | O Poor |
| 1. In case you get sickness, do you withdraw for the work | O yes | O No |
| 1. How much you spend to cure you sickness | O Specify…………………………………………………… | |
| 1. How many days you lose from illness over 6 months period | O Specify…………………………………………………… | |

1. Environmental management

| 1. Method of liquid waste treatment before draining | O Clarifier | O Treated pond | O Others  .................. | O None |
| --- | --- | --- | --- | --- |
| 1. Liquid waste draining site | O SLH area | O Community | O Stream | O Other..... |
| 1. Method to treat the feathers | O bury | O burn | O sell | O Other………………… |
| 1. Method to treat the feces | O dry | O discard | O other  …………………. | O None |
|  | O bury | O composting |  |  |
| 1. Method to treat solid waste other than feces (feathers and others) | O burn | O discard | O other  …………………. | O None |
| 1. Human exposure to the waste: Are people in contact with wastewater and feces? | O Yes | O No |  |  |
| 1. Reuse of the waste: Do people use wastewater for irrigation and feces for fertilising field? | O Yes | O No |  |  |
| 1. Waste water draining site | O SLH area | O Community | O Other..... | O None |

10. Socio-economics

| 1. Did you borrow money to conduct your operations between July 2010 and June 2011? | O Yes  How much? …………….  From where? ……………….. | O No |  |  |
| --- | --- | --- | --- | --- |
| 1. After borrowing, do you get enough funds to conduct your operations? | O Yes | O No |  |  |
| 1. Did you sell goods on consignment? | O Yes | O No |  |  |
| 1. What percentage of your sales are made on consignment? | O Yes | O No |  |  |
| 1. On average, how long is the period of consignment? | O ------------(what is the unit here?) | O No |  |  |
| 1. Has your business ever had contracts for the production of carcass? | O Yes  what percentage of your total carcass sales? …………… | O No |  |  |
| 1. Your business ever had contracts for procurement of live poultry? ………………………. | O Yes  what percentage of your total procurement of live poultry? ……………… | O No |  |  |
| 1. How do you consider the profitability of your … activities during the period from July 2009 to June 2010 | O Good | O Fair | O Poor |  |
| 1. How do you consider the profitability of your … activities during the period from July 2010 to June 2011? | O Good | O Fair | O Poor |  |
| 1. If the profitability ranking changed between last year and this year, what is the main reason for the change in the profitability ranking of your business activities? | 1. Sale price | 2. Purchase price | 3. Volume of trade | 4. Competition level |
|  | 5.Labor costs | 6. Intereste rate | 7. Technology level | 8. Other |
| 11. Percentage of slaughtering business in total income? | ……………………. |  |  |  |

| **Topic** | Strongly agree | Agree | Indifferent | Disagree | Strongly disagree |
| --- | --- | --- | --- | --- | --- |
| 1. The sick poultry can transmit the disease to human |  |  |  |  |  |
| 1. The poultry which look healthy can transmit the disease to human |  |  |  |  |  |
| 1. The dead poultry can transmit the disease to human |  |  |  |  |  |
| 1. The pathogen can contaminate to the slaughterhouse area |  |  |  |  |  |
| 1. The pathogen can spread into the environment |  |  |  |  |  |
| 1. The pathogen can be eliminate |  |  |  |  |  |
| 1. The slaughterhouse cleaning measures is important |  |  |  |  |  |
| 1. The withdrawal period for slaughterhouse is important |  |  |  |  |  |
| 1. Inspection the chicken before slaughter is important |  |  |  |  |  |
| 1. Inspection the meat after slaughter is important |  |  |  |  |  |
| 1. The unqualified chicken should not be slaughtered |  |  |  |  |  |
| 1. The unqualified meat should not be consumed |  |  |  |  |  |
| 1. The workers in slaughterhouse can protect themselves from diseases |  |  |  |  |  |
| 1. The workers in slaughterhouse should have zoonotic prevention knowledge |  |  |  |  |  |

1. **Zoonotic aspect perception**
