# Supplementary material for: An integrative approach to enhancing small-scale poultry slaughterhouses by addressing regulations and food safety in northern -Thailand
Source: Infect Dis Poverty. 2014 Dec 5;3:46. doi: 10.1186/2049-9957-3-46 (PMC4322817; doi:10.1186/2049-9957-3-46)
Supplement: Supplementary file 3 — Additional file 3: Check list for slaughterhouses. (DOCX 17 KB) [file 40249_2014_86_MOESM3_ESM.docx]

**Check list for slaughterhouses**

**Slughter house name…………………………………………………………ID……………………….**

**Date…….…./……………../………...Recorder…………………………………………………………**

|  | **Subject** | **Yes** | **No** |
| --- | --- | --- | --- |
| **1** | **Place** |  |  |
|  | **1.1 Location** |  |  |
|  | Located far away from educational place, temple, hospital, dormitory |  |  |
|  | Avoid noise |  |  |
|  | Located far away from community (more than 1 km) |  |  |
|  | **1.2 building** |  |  |
|  | The floor is concrete or non water –absorbed material and easy to clean |  |  |
|  | The wall is build by plain materials and easy to clean |  |  |
|  | The separated room for pick up the equipments |  |  |
|  | The equipments which contact with the meat (knife) are plain materials, no rust, easy to clean, and durable for disinfectant. |  |  |
|  | The fix instruments are separated from the wall (>30 cm) for easy to clean |  |  |
|  | There is sufficient light (>200 Lux) |  |  |
|  | There is sufficient air flow ( ventilator should more than 1/10 of the area) |  |  |
| **2** | **Sanitation** |  |  |
|  | 2.1 There are suitable methods to prevent the meat contacted with the dirty |  |  |
|  | 2.2 Waste management  -there is places for separsating the waste  -There is the method to prevent the odor, the noise which annoyed and harm to the people around the SLH |  |  |
|  | 2.3 Cleaning and disinfection  -The equipments are cleaned before and after use.  -There is schedule for clean and disinfection the SLH (Every week)  -Disinfectants are proved to use in the food industry  -The water and the ice used in the SLH are clean. |  |  |
|  | 2.4 Pest control  -There is schedule to eradicate the rodents, birds, insects  -The pesticide used in the SLH is proved to used in food industry |  |  |
|  | 2.5 Waste water management  -There is waste water management system  -The waste water does not drain to the community |  |  |
| **3** | **Slaughtering processes** |  |  |
|  | 3.1 The poultry are inspected before slaughter |  |  |
|  | 3.2 The meat are inspected after slaughter |  |  |
|  | 3.3 There is method to destroy the disqualified meat |  |  |
| **4** | **Meat storage management** |  |  |
|  | 4.1 There is the method to control the temperature of the meat before sending to the market |  |  |
|  | 4.2 There is method to prevent the meat from contamination |  |  |
